# Early nutritional management and risk of neonatal bronchopulmonary dysplasia: a systematic review and mata analysis

**DOI:** 10.1186/s13052-025-01929-5

**Published:** 2025-03-24

**Authors:** Jiayi Yang, Hua Mei, Mengyue Huo, Yuheng Zhang, Chun Xin

**Affiliations:** Department of Neonatology, The Affiliated Hospital of Inner Mongolia Medical University, Hohhot, China

**Keywords:** Bronchopulmonary dysplasia, Nutritional management, Fluid management, Meta-analysis

## Abstract

This study examines the relationship between early postnatal nutrition, fluid management in preterm infants, and the risk of bronchopulmonary dysplasia (BPD). A comprehensive review was conducted across multiple databases, including PubMed, Embase, Cochrane Library, Web of Science, and major Chinese databases, covering studies up to 31 August 2024. Case-control and cohort studies on nutritional management and BPD were analyzed using meta-analysis in Stata 15.0. The study is registered on Prospero (CRD42023484951). A total of 11 studies involving 1,826 infants (894 with BPD, 931 controls) were included. Findings showed significantly lower calorie intake in the BPD group during the first postnatal week (Mean Difference = -6.20, 95% CI: -8.91 to -3.48, *P* < 0.05). While fluid intake differences were not statistically significant (Mean Difference = 5.31, 95% CI: -0.57 to 11.19, *P* = 0.077), the BPD group had lower protein (Mean Difference = -0.13, 95% CI: -0.22 to -0.04, *P* < 0.05), lipid (Mean Difference = -0.39, 95% CI: -0.49 to -0.299, *P* < 0.05), and carbohydrate intake (Mean Difference = -0.74, 95% CI: -0.95 to -0.54, *P* < 0.05). Time to full enteral nutrition was also prolonged in the BPD group (Mean Difference = 11.23, 95% CI: 9.68–12.78, *P* < 0.05). These results suggest that inadequate early nutrition may increase BPD risk. Optimizing nutritional and fluid management, alongside respiratory support, is essential for prevention.

**Supplementary Information:**

The online version contains supplementary material available at 10.1186/s13052-025-01929-5.

## Introduction

Bronchopulmonary dysplasia (BPD) stands as chronic respiratory sequelae, intricately woven by the interplay of multiple determinants, significantly impacting the prognosis and long-term quality of life for neonates [1]. Several studies have shown [2–3] that nutritional support in the early postnatal period of preterm infants, especially in the first postnatal week, is negatively correlated with the incidence of BPD, that calories in the first postnatal week of ultra-low birth weight infants with BPD are significantly lower than those of non-BPD children, and that low-calorie intake is associated with the severity of BPD, and that preterm infants need adequate nutritional support in the initial postnatal period to achieve optimal growth and development. This is because the first week of life is a critical period for lung development. The lung structures of preterm infants are not yet fully mature, and adequate nutrition helps to support alveolar formation and improved lung function. The judicious administration of appropriate nutrition during the initial postnatal period for preterm infants transcends mere growth facilitation. It emerges as a crucial catalyst in steering the comprehensive development and maturation of the respiratory system, offering essential fortification for lung development and thereby mitigating the vulnerability to BPD incidence [4–5]. This study employs a Meta-analysis framework, systematically gathering pertinent clinical research literature to provide more detailed aspects of nutritional requirements and their impact on BPD and scrutinize the intricate relationship between nutritional and fluid management and the propensity for BPD development in the first week of life among preterm infants. The ultimate goal is to furnish an evidence-based foundation for the nuanced nutritional management of neonates with BPD during their formative early stages of life.

## Data and methods

We conducted this study following the Preferred Reporting Items for Systematic Reviews and Meta-Analyses flow diagram (PRISMA 2020) guidelines. (Supplemental Table 1) [6]. 

### Literature search strategy

We searched PubMed, Embase, Web of Science, Cochrane Library, China Knowledge Network Infrastructure (CNKI), Wanfang, and China Biomedical Literature Database, as well as reading relevant reference lists and trial registries. The temporal scope of this search spanned from the inception of each respective database to 31 August 2024. The search strategy employed a judicious fusion of subject-specific terminology and unconstrained free terms to ensure an exhaustive and nuanced retrieval of pertinent literature. The search strategies for this meta-analysis were as follows: (Bronchopulmonary dysplasia OR pulmonary disease OR Lung dysplasia) AND (Nutrition OR Enteral nutrition OR Parenteral nutrition OR Liquid OR Calorie OR Energy). The two authors (Yang and Mei) searched the database independently and then imported it to the Note Express software for further analysis.

### Literature inclusion criteria

1. Study subjects: birth gestational age < 32 weeks and birth weight < 1500 g;
2. Type of study: I. The literature included case-control or cohort studies; II. The observation and control groups were BPD and non-BPD preterm infants, respectively. Data can be provided as mean and standard deviation (SD), or can be converted to 95% CI of mean and standard deviation or Interquartile Range (IQR). The data conversion method in this article uses an online calculator developed by the Institute of Mathematics, Hong Kong Baptist University. (http://www.math.hkbu.edu.hk/~tongt/papers/median2mean.html)
3. The literature describes the diagnostic criteria for BPD [7] and unified as NICHD (National Institutes of Health Consensus Definition) diagnostic criteria;
4. Clear values of enteral and parenteral calorie, fluid intake including daily milk and intravenous fluids, and macronutrient (protein, fat, and carbohydrates) intakes were required.

### Literature exclusion criteria

1. Repeated articles.
2. Incomplete raw data or no further information could be obtained.
3. Case reports, reviews, letters, dissertations, and unpublished articles.

### Literature screening and data extraction methods

Independently, the two researchers (Yang and Mei)collected the database, scrutinizing titles and abstracts to eliminate literature not meeting the inclusion criteria. Those potentially meeting the criteria underwent a comprehensive reading. The two researchers meticulously cross-verified their screening outcomes, ensuring consensus on the inclusion of literature. Any disparities that emerged during this process were meticulously adjudicated by a third researcher. The gleaned information from the ultimately selected literature encompassed key details such as the primary author, publication date, sample size, gestational age, birth weight, feeding protocols, adherence to nutritional guidelines, and specific values pertaining to calorie, fluid, and macronutrient intake during the postnatal week.

### Literature quality assessment

To assess the scholarly studies’ quality, the Newcastle-Ottawa Scale (NOS) [8] was employed. The NOS incorporates three fundamental domains: selection, comparability, and outcomes, featuring eight criteria and a top score of nine. A study is deemed high quality if it scores above six on this scale. Throughout the quality evaluation of academic literature, any uncertainties were thoroughly deliberated with a third researcher for resolution.

### Statistical analysis

The statistical analysis encompassed a meta-analysis of data utilizing the statistical software version Stata 15.0. Employing mean difference and 95% confidence interval(95%CI) as the effect analysis statistic for continuous variables, we assessed the heterogeneity of the included data. The I^2^ test was used to judge heterogeneity, I^2^ > 50% showed that the heterogeneity difference was statistically significant, random effect model was used for meta-analysis. I^2^ < 50% showed that heterogeneity difference had no statistical difference, and a fixed effect model was used for meta-analysis. A significance level of *p* < 0.05 was deemed statistically significant.

## Results

### Results of literature search

**Fig. 1:**
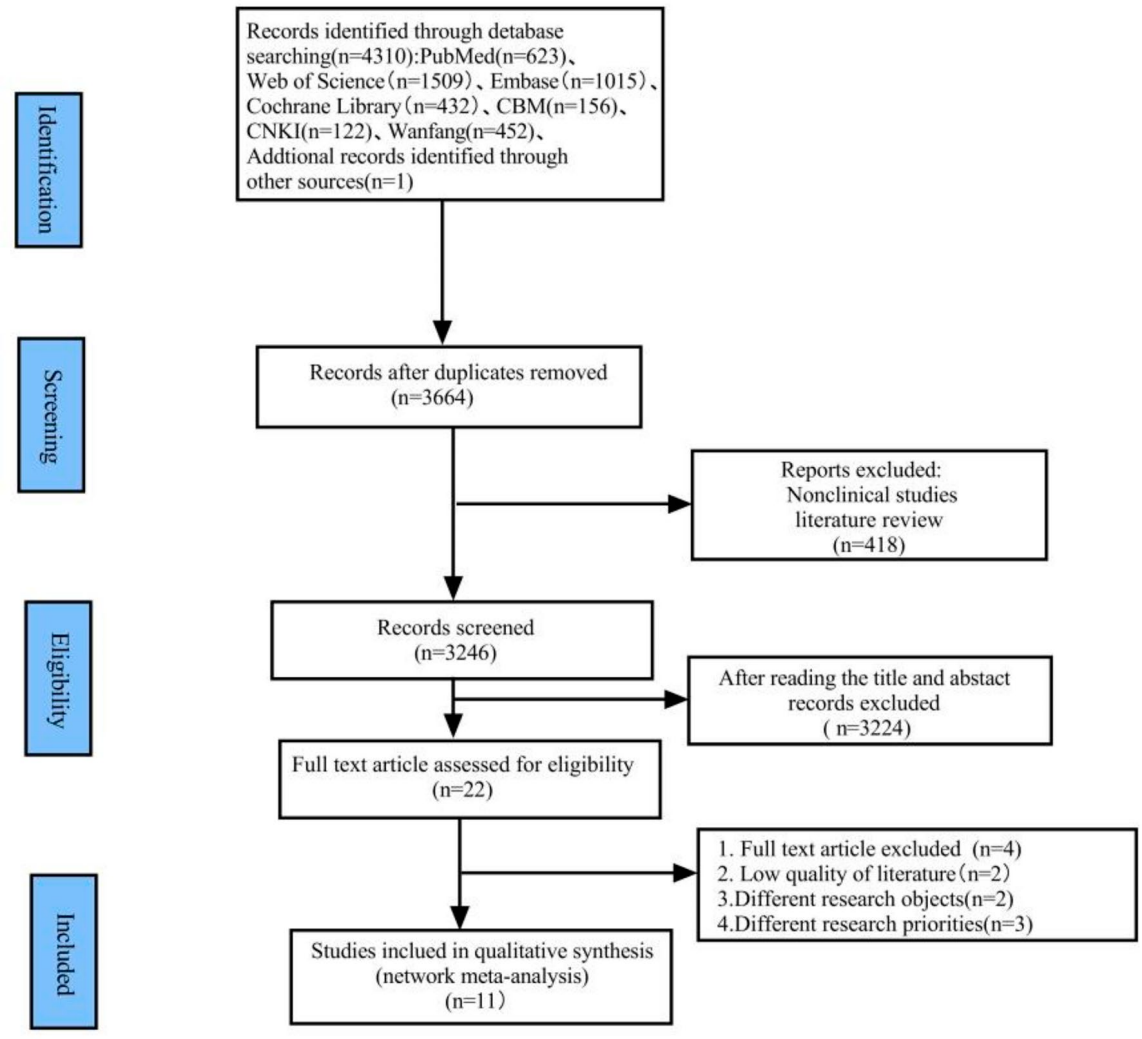
Systematic progression of the literature search

Out of a total of 4310 relevant articles identified, 12 articles were finally selected for this study through a step-by-step screening process, as visually depicted in Fig. 1.

### Basic characteristics and quality assessment of included studies

The comprehensive assessment of the 11 incorporated research articles involved 1825 participants, encompassing 894 subjects diagnosed with BPD and 931 without BPD. The inclusive selection comprised four case-control studies [9–12] and six cohort studies [13–19]. Detailed information, including the essential characteristics and evaluation scores based on the NOS, is presented in Tables 1 and 2.

**Table 1:** Characteristics of the included studies

Study	Methods	BPD/Non-BPD			Country	Feeding methods	Nutrition guidelines	Main outcomes
		Sample	Gestational age (x ± s, weeks)	Birth weight (x ± s, g)				
Lin 2024 [9]	Retrospective cohort study	250/250	29.5 ± 1.3/29.5 ± 1.3	1107 ± 258, 1324 ± 261	China	Enteral, parenteral	Not available	Calorie and fluid intake at 1 week postnatal period
Xu 2023 [10]	Retrospective case-control study	79/73	29.85 ± 1.48/29.94 ± 1.51	1173 ± 267, 1275 ± 235	China	parenteral	Not available	Calorie, fluid, and macronutrient intake values at 1 week postnatal period
Lin 2022 [11]	Retrospective cohort study	43/91	26 (25;27 + 2), 27 + 3 (26 + 5;28 + 4)	813 ± 120, 936 ± 123	China	Enteral, parenteral	ESPHGAN	Calorie, fluid, and macronutrient intake values at 2 week postnatal period
Li 2022 [12]	Retrospective case-control study	60/97	29.1(26.7–31.7), 30.2(27.1–31.9)	1215 (790–1490), 1320 (1020–1490)	China	Not available	Not available	Fluid intake during the postnatal week
Milanesi 2021 [13]	Prospective cohort study	16/62	26.8 ± 2.5, 29.4 ± 1.9	851 ± 244, 1264 ± 343	Brazil	Enteral, parenteral	ESPHGAN	Calorie, fluid, and macronutrient intake in the first month of life
Xu 2021 [14]	Retrospective case-control study	86/68	28.35 ± 1.55, 30.12 ± 1.23	1050.91 ± 190.6, 1205.88 ± 195.83	China	Enteral, parenteral	2015 Canadian VLBWI Feeding Guidelines + 2013 Chinese Neonatal Nutritional Support Clinical Application Guidelines	Calorie, fluid, and macronutrient intake values at 2 week postnatal period
Ding 2020 [15]	prospective cohort study	30/42	29.76 ± 1.54, 30.28 ± 1.2	1305.00 ± 315.76, 1391.67 ± 231.81	China	Enteral, parenteral	Not available	Calorie and fluid intake on days 3, 7, 14, and 28 postnatally
Jebawi 2020 [16]	retrospective cohort study	151/75	24.7 ± 1.7, 26.8 ± 2.0	678 ± 154, 837 ± 129	USA	Enteral, parenteral	NICU guidelines	Calorie, fluid, and macronutrient intake values at 1 week postnatal period
Malikiwi 2019 [17]	Retrospective case-control study	33/33	25.6 ± 1.1, 26.4 ± 1.1	748.6 ± 127.1, 831.0 ± 102.9	Australia	Enteral, parenteral	Not available	Calorie, fluid, and macronutrient intake in the first month of life
Alshaikh 2017 [18]	retrospective cohort study	125/108	25.9 ± 1.4, 26.9 ± 1.2	832 ± 191, 1028 ± 206	Canada	Enteral, parenteral	Not available	Calorie, fluid, and protein intake values at 1 week postnatally
Lehtinen 2017 [19]	prospective cohort study	21/32	28.6407 ± 7.1553, 30.0683 ± 6.9843	1018.025 ± 663.8531, 1095.9766 ± 636.3479	Finland	Enteral, parenteral	Not available	Calorie, and fluid intake at 1-week postnatal period

**Table 2:** Newcastle-Ottawa scale for assessing the quality of studies in meta-analysis

Study quality of cohort studies	Selection				Comparability	Outcome			
**Author**	**Representativeness of the exposed cohort**	**Selection of the non-exposed cohort**	**Ascertainment of exposure**	**Demonstration that outcome of interest was not present at start of study**	**Comparability of cohorts on the basis of the design or analysis**	**Assessment of outcome**	**Was follow-up long enough for outcomes to occur**	**Adequacy of follow up of cohorts**	**Total scores**
Lin 2024 [9]	★	★	★	★	★★		★		7
Lin 2022 [11]	★	★	★	★	★★		★		7
Milanesi 2021 [13]	★	★	★	★	★		★	★	7
Ding 2020 [15]	★	★	★	★	★	★	★	★	8
Jebawi 2020 [3]	★	★	★	★	★	★	★	★	8
Alshaikh 2017 [18]	★	★	★	★	★		★	★	7
Lehtinen 2017 [19]	★	★	★	★	★		★	★	7
**Study quality of case-control studies**	**Selection**				**Comparability**	**Exposure**			**Total scores**
**Author**	**Adequate definition of cases**	**Representativeness of the cases**	**Selection of controls**	**Definition of controls**	**Control for important factor**	**Ascertainment of exposure**	**Same method of ascertainment for cases and controls**	**No responserate**	**Scores**
Xu 2023 [10]	★	★		★	★★	★	★	★	7
Li 2022 [12]	★	★	★	★	★	★	★	★	8
Xu 2021 [14]	★	★		★	★★	★	★	★	7
Malikiwi 2019 [17]	★	★	★	★	★	★	★	★	8

### Meta-analysis results

#### Calorie intake

##### Meta-analysis

**Fig. 2:**
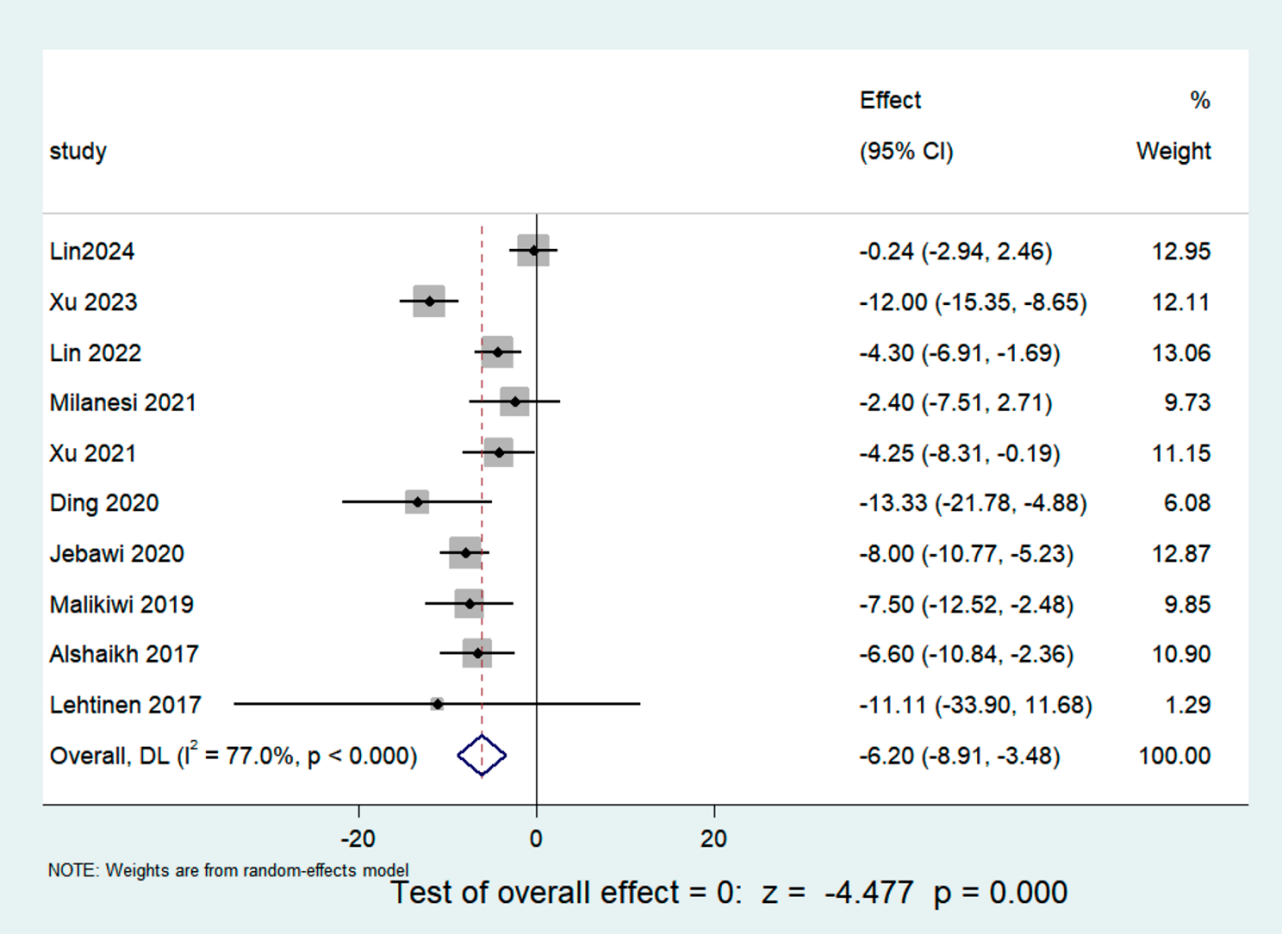
Comparison of calorie intake values at postnatal week 1

A collective of 10 papers incorporated in this analysis [9, 11–19] presented the total calorie intake data for children during the postnatal week, collectively presenting data on calorie intake throughout the initial postnatal week. The dataset encapsulated 1668 children and underwent heterogeneity testing, revealing an I2 value of 77%, indicative of substantial heterogeneity among the selected papers. Consequently, a random-effects model was applied. The meta-analysis results demonstrated a pooled Mean Difference of -6.20 with a 95% CI ranging from − 8.91 to -3.48. The statistical significance was affirmed with a z-score of -4.477 and a P-value less than 0.05, signifying that the BPD group exhibited a diminished calorie intake in the initial week of life compared to the non-BPD group, as visually depicted in Fig. 2.

##### Sensitivity analysis of calorie intake

**Fig. 3:**
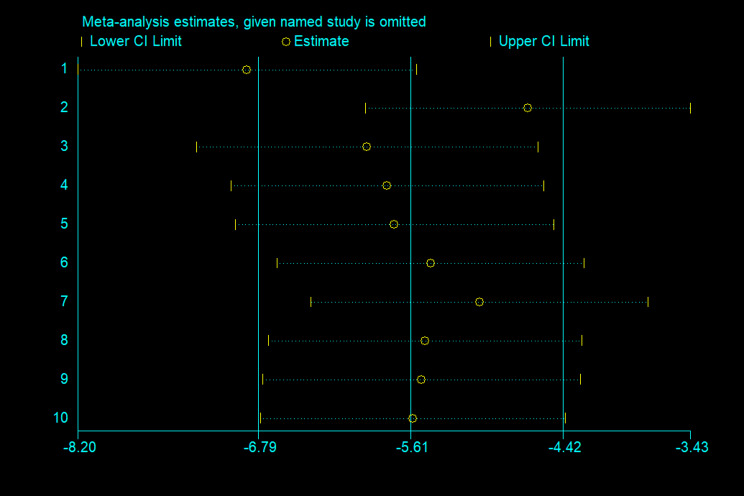
Sensitivity analyses of Calorie intake

In this study, sensitivity analyses were also performed on 10 papers, with Lin2024 as the main source of heterogeneity, which may be related to different individualised treatments for different preterm babies, as shown in Fig. 3.

##### Subgroup analysis of calorie intake

Meta-analysis results presented high heterogeneity in Calorie intake for the primary outcome indicators, and subgroup analyses based on birth weight, study area, study method, and different feed methods did not reveal any source of heterogeneity, is presented in Table 3.

**Table 3:** Calorie intake subgroup analysis

Group	Sample size	Heterogeneity test		Effect model	OR (95%CI)	*P*
		I^2^	*P*			
Birth weight						
< 1000 g	4	0%	0.596	fixed	-5.13 (-6.95,-3.31)	0.000
> 1000 g	6	86.4%	< 0.01	Random	-5.96 (-7.52,-4.40)	0.000
Country						
China	6	86.7%	< 0.01	Random	-5.50 (-6.84, -4.16)	0.000
Other	4	0%	0.723	fixed	-5.98 (-8.50, -3.46)	0.000
Methods						
case-control study	4	74.3%	0.009	Random	-8.70 (-10.96,-6.45)	0.000
Cohort study	6	71.3%	0.004	Random	-4.43 (-5.82,-3.03)	0.000
Feeding Methods						
Enteral, parenteral	9	65.4%	0.003	Random	-4.69 (-5.96,-3.43)	0.000
Parenteral	1	0%	< 0.01	Random	-12 (-15.35,-8.65)	0.000

#### Fluid intake

##### Meta-analysis

**Fig. 4:**
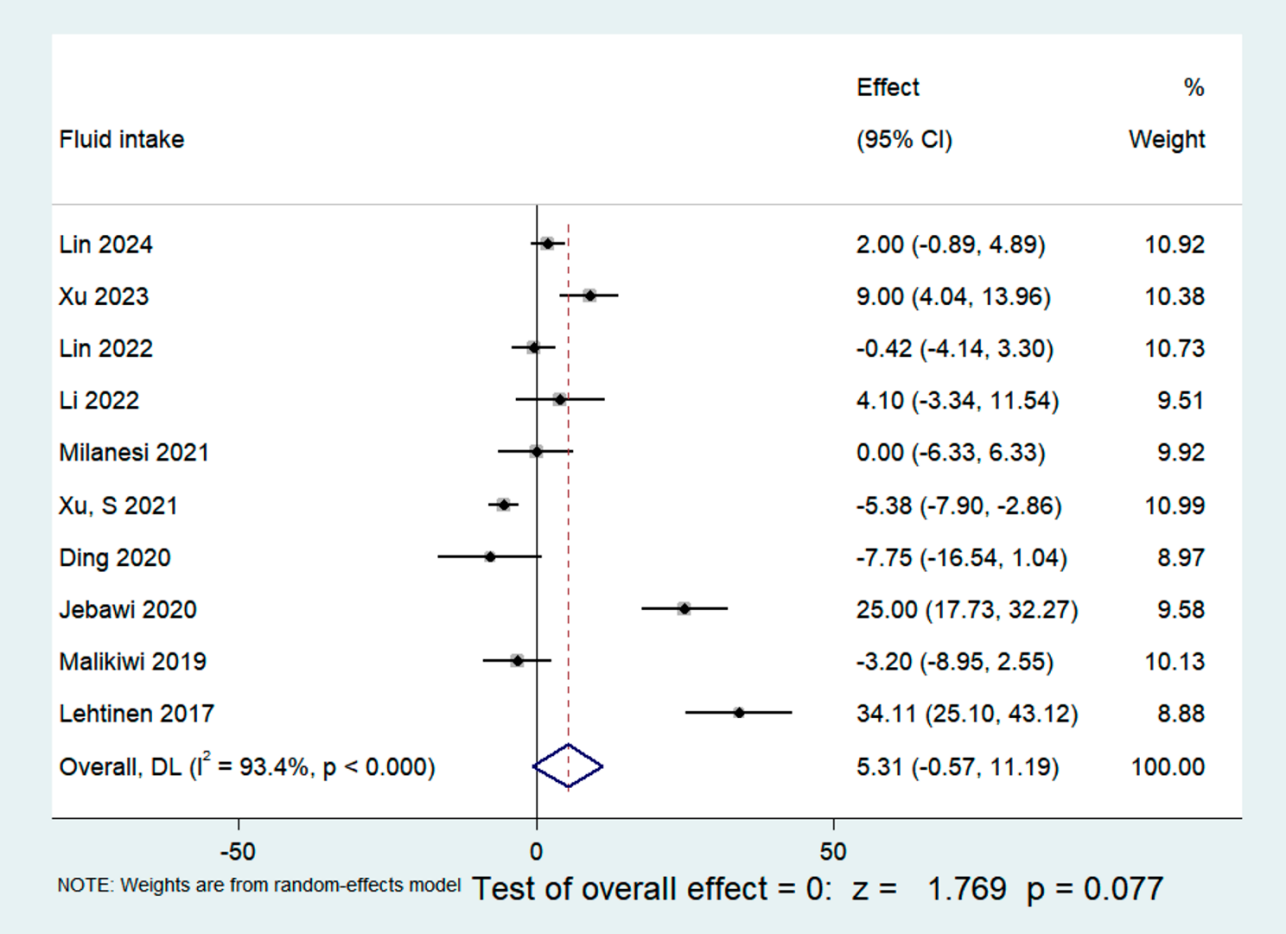
Comparison of fluid intake values at postnatal week 1

10 publications were included, collectively presenting data on fluid intake throughout the initial postnatal week. The dataset encapsulated 1592 children and underwent heterogeneity testing, revealing an I^2^ value of 93.4% and a P-value less than 0.01, indicative of notable heterogeneity among the selected papers. Consequently, a random-effects model was applied. The meta-analysis results unveiled a pooled Mean Difference of 5.31, accompanied by a 95% CI spanning from − 0.57 to 11.19. Despite a statistical significance with a z-score of 1.769, the P-value of 0.077 exceeded 0.05. None of the observed distinctions attained statistical significance, suggesting that infants with BPD did not exhibit elevated fluid intake during the initial week of life compared to their non-BPD counterparts, as illustrated in Fig. 4.

##### Sensitivity analysis of fluid intake

**Fig. 5:**
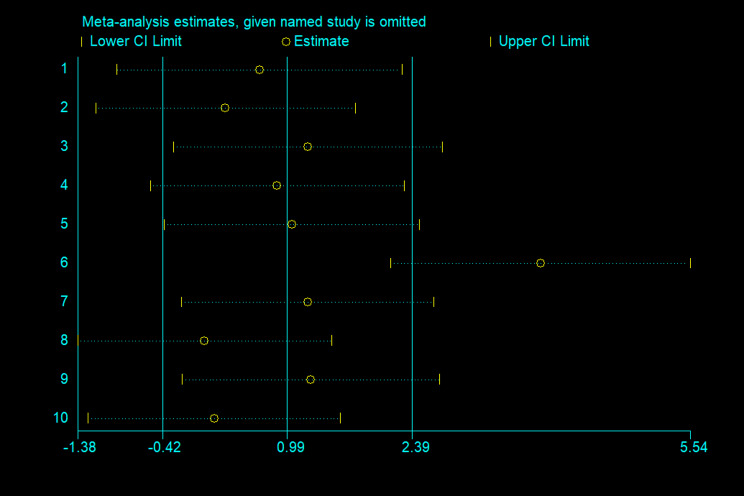
Sensitivity analyses of Fluid intake

Sensitivity analyses were also performed on the nine papers in this study, and Xu 2021 caused greater interference with the results of this meta-analysis, as illustrated in Fig. 5.

##### Subgroup analysis of fluid intake

Meta-analysis results presented high heterogeneity in fluid intake for the primary outcome indicators, and subgroup analyses based on birth weight, study area, study method, and different fluid loads did not reveal any source of heterogeneity, and none of the differences in the results of the subgroups were with statistical significance (all P-values > 0.05), is presented in Table 4.

**Table 4:** Fluid intake subgroup analysis

Group	Sample size	Heterogeneity test		Effect model	OR (95%CI)	*P*
		I^2^	*P*			
Birth weight						
< 1000 g	4	93.1%	< 0.01	Random	5.09 (-5.51,15.69)	0.346
> 1000 g	6	94.5%	< 0.01	Random	5.56 (-2.49, 13.61)	0.176
Country						
China	6	85.8%	< 0.01	Random	0.40 (-4.14,4.95)	0.863
Other	4	95.9%	< 0.01	Random	13.75 (-3.39, 30.89)	0.116
Methods						
case-control study	4	89.5%	< 0.01	Random	0.92 (-6.47,8.32)	0.807
cohort study	6	94.5%	< 0.01	Random	8.47 (-0.81,17.75)	0.074
Fluid intake (mL/kg·d)						
> 150	3	94.2%	< 0.01	Random	7.23 (-11.41,25.88)	0.447
120–150	5	95.3%	< 0.01	Random	6.05 (-3.16, 15.26)	0.198
< 120	2	0%	0.574	Random	0.000 (-6.33, 6.33)	0.218

#### Macronutrient intake

##### Protein intake

**Fig. 6:**
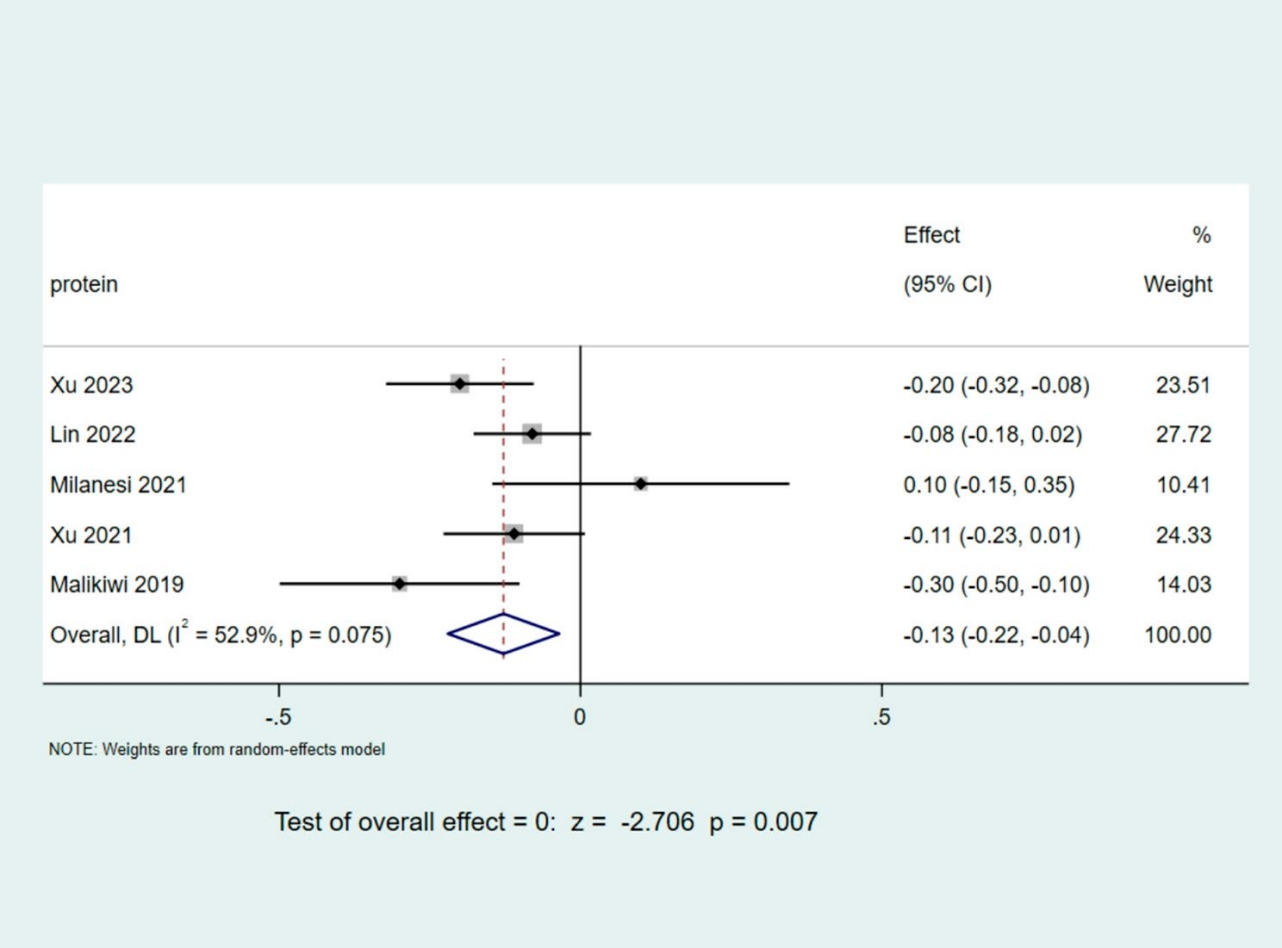
Comparison of protein intake values at postnatal week 1

A total of 5 studies [9, 12, 14, 15] reported the value of protein intake during the postpartum week, and a total of 584 children were included. The heterogeneity examination, with an I^2^ value of 52.9% and a P-value of 0.075, indicated a mild heterogeneity among the compiled literature, staying within an acceptable range. Consequently, a random effects model was implemented. The meta-analysis outcomes demonstrated a statistically significant Mean Difference of -0.13, accompanied by a 95% CI ranging from − 0.22 to -0.04. With a z-score of -2.706 and a P-value less than 0.05, the results were deemed statistically significant. This implies that, during the initial week of life, the protein intake of infants with BPD was markedly lower than that of their non-BPD counterparts. A detailed depiction is presented in Fig. 6.

##### Lipid intake

**Fig. 7:**
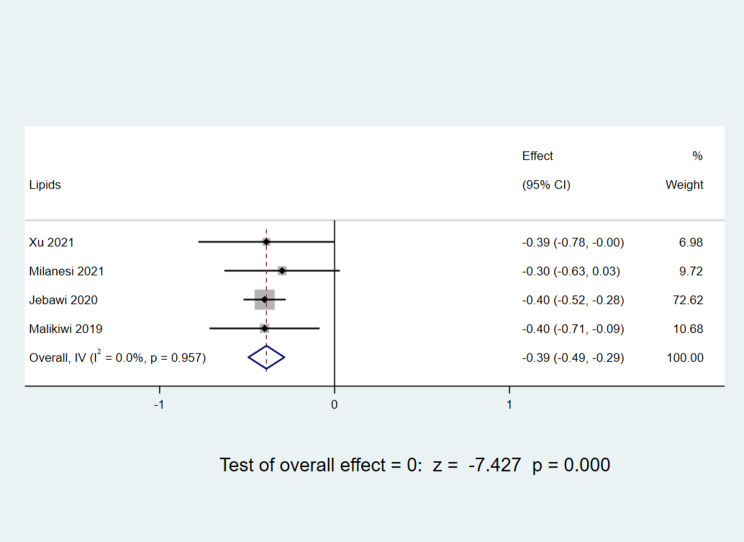
Comparison of lipid intake values at postnatal week 1

Four distinct studies [11, 12, 15, 17] reported lipid intake during the postnatal week, encompassing a total of 584 infants. Post heterogeneity assessment, yielding an I^2^ value of 0% and a P-value of 0.957, indicating an absence of substantial heterogeneity within the selected literature, a fixed-effect model was employed. The meta-analysis results revealed an Mean Difference value of -0.39, with a 95% CI ranging from − 0.49 to -0.299. With a z-score of -7.427 and a P-value below 0.05, the outcomes were deemed statistically significant. This underscores that, in the initial week of life, infants diagnosed with BPD exhibited significantly lower lipid intake compared to their non-BPD counterparts, as illustrated in Fig. 7.

##### Carbohydrate intake

**Fig. 8:**
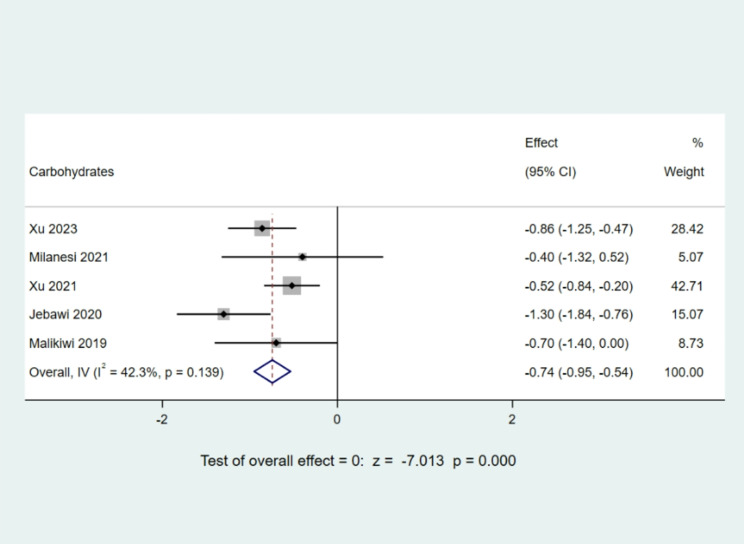
Comparison of carbohydrate intake values at postnatal week 1

The cumulative data from five studies [9, 11, 12, 15, 17] detailing carbohydrate intake in the postnatal week included a total of 676 children. Following the heterogeneity examination, with an I^2^ value of 42.3% and a P-value of 0.139, indicating an absence of notable heterogeneity within the chosen literature, a fixed-effect model was employed. The Meta-analysis outcomes revealed an Mean Difference value of -0.74, accompanied by a 95% CI ranging from − 0.95 to -0.54. With a z-score of -7.013 and a P-value below 0.05, the results were considered statistically significant. This underscores that, during the initial week of life, children diagnosed with BPD exhibited significantly lower carbohydrate intake compared to their non-BPD counterparts, as depicted in Fig. 8.

#### Time to reach complete enteral feeding

**Fig. 9:**
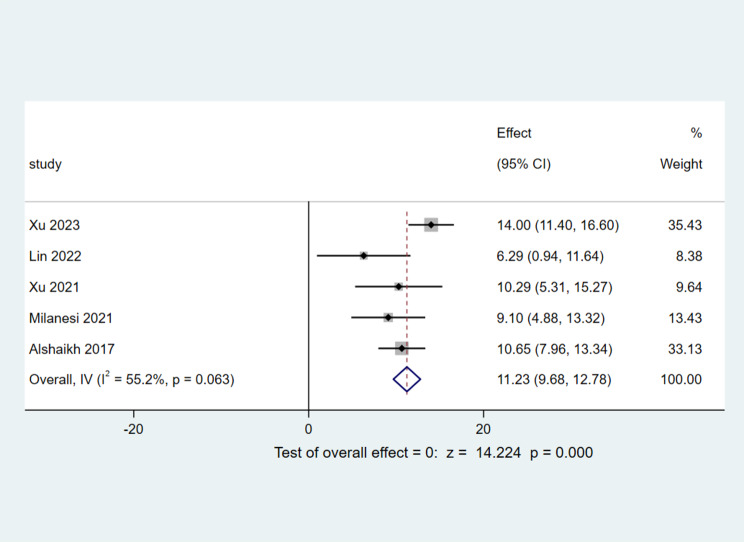
Comparison of the duration of up to full enteral feeds

A total of five studies [9, 11, 14, 15, 18] reported the time required to reach complete enteral feeding in preterm infants, encompassing a total of 751 infants, after the heterogeneity test, (I^2^ = 55%, *p* = 0.06), signifying heterogeneity among the chosen literature, but it was within acceptable ranges. Consequently, a fixed-effect model was applied. The Meta-analysis results revealed an Mean Difference value of 11.23, accompanied by a 95% CI ranging from 9.68 to 12.78. With a z-score of 14.22 and a p-value below 0.05, the outcomes were deemed statistically significant. This suggests that, in comparison to the non-BPD group, infants diagnosed with BPD exhibited a significantly prolonged time to achieve complete enteral feeding, indicating that BPD may have an adverse impact on this physiological process, as depicted in Fig. 9.

## Discussion

In the contemporary era, marked by advancements in perinatal medicine, the survival rates of infants afflicted by BPD have witnessed an upward trajectory. Nonetheless, BPD persists as a pervasive challenge, standing out as one of the most prevalent and consequential sequelae affecting preterm infants. The ramifications extend beyond the individual to cast a substantial burden on both families and society at large. A burgeoning body of research underscores the pivotal role of early nutritional support in mitigating the risk of BPD among preterm infants. This investigation seeks to unravel the intricate interplay between BPD occurrence and the nuanced domains of nutritional and fluid management. The overarching aim is to refine and elevate the standards of nutritional and fluid management for the betterment of preterm infants.

This investigation reveals a noteworthy distinction in caloric provisioning between the non-BPD and BPD groups during the initial postnatal week. The findings underscore infants with BPD exhibited a diminished calorie intake in the first week of life compared to those without BPD, suggesting that increasing calorie intake in preterm infants may promote protection against BPD. Notably, children grappling with BPD exhibit an augmented demand for calories attributable to heightened metabolic requisites and increased respiratory workload. Consequently, a keen focus on caloric supply is imperative. Experimental animal models have corroborated that caloric restriction diminishes alveolar count and reduces the effective alveolar surface area [20]. Additionally, Ehrenkranz et al. [2] established a correlation wherein a 2% reduction in BPD prevalence per 1 Kca/kg-d increase in weekly total energy intake was observed in neonates. A prospective study by Klevebro et al. [21] also found that for every 10 kcal/(kg-d) increase in energy intake from postnatal day 7 to 27, the risk of BPD was reduced by 9% (*P* = 0.029). Early active nutrition may strengthen the degree of tolerance to disease by increasing the preterm infants’ resistance to infection and oxidative stress damage [22]. Therefore high calorie intake is essential to prevent the development of BPD in preterm infants.

Body fluids, integral conveyors of nutrients and metabolites, wield significant influence on postpartum BPD genesis. Deviations in fluid supply during this critical period can precipitate abnormal fluid status, contributing to BPD onset. Striking a balance is paramount; fluid restriction impairs energy intake, fostering malnutrition and stunted growth [23]. Conversely, elevated fluid volume may culminate in pulmonary edema, compromised lung compliance, heightened airway resistance, impaired gas exchange, and escalated reliance on mechanical ventilation [24–26]. Matsushita et al. [27]found that fluid overload in very preterm infants during the first 72 h of life was associated with higher mortality and longer periods of mechanical ventilation. Intriguingly, our study renders fluid loading and BPD development statistically non-significant. This may be related to the inconsistency in the timing of fluid management between studies, as some studies have shown [28], that fluid restriction on postnatal days 3–7 also failed to reduce the incidence of BPD. Therefore, it is important to avoid fluid overload during the first 72 h of life to reduce pulmonary edema and to avoid inadequate nutritional intake due to excessive fluid restriction later in life.

Proteins are involved in a range of physiological processes critical to neonatal development as cellular structural components, and preterm infants have greater protein requirements than term infants [29]. Emerging research posits that protein deficiency in preterm infants intensifies lung tissue oxidation, precipitating alveolar thinning and hastening BPD progression [30]. Our study corroborates a statistically significant difference in protein provisioning during the first postnatal week between BPD and non-BPD groups. Underscoring the imperative of prioritizing heightened protein intake during early postnatal stages.

Our study underscores the salutary role of fat in providing ample calories, curbing oxidative stress, and bolstering tissue growth. Clinical trials demonstrate that neonates administered parenteral nutrition infused with fish oil fat emulsion manifest a reduced likelihood of severe BPD [31]. Furthermore, fat emulsions exhibit the potential to attenuate inflammatory responses and enhance immune function [32]. Ensuring adequate calorie intake to diminish BPD risk mandates the early incorporation of lipids into intravenous nutritional solutions [33]. Our study echoes this sentiment, elucidating that fat milk supply in the non-BPD group significantly surpasses that in the BPD group during the postnatal week. This observation reinforces the pivotal role of fat milk in furnishing preterm infants with requisite calories and reducing BPD susceptibility. Consequently, vigilant attention to fat milk intake in intravenous nutrition is advocated.

Carbohydrates stand as the primary energy source for preterm infants during the early postnatal phase, with glucose serving as the principal substrate for carbohydrate utilization. Glucose, a ubiquitous metabolic fuel, caters to the energy demands of vital organs. Al-Jebawi et al. discerned a significant reduction in carbohydrate intake within the moderate to severe BPD cohort during the initial week of life compared to the non-BPD and mild BPD groups [17]. Thiess [34] further affirms that carbohydrate intake fosters pulmonary development, aligning with our study’s findings. Emphasis on meticulous attention to carbohydrate intake in preterm infants is warranted. Simultaneously, a heightened carbohydrate load may elevate basal oxygen consumption and carbon dioxide production, posing pulmonary stress risks [35]. Careful control of the glucose supply rate is crucial, ensuring a per-minute glucose administration of less than 12 mg/kg to avert undue speed-associated complications.

Prolonged reliance on intravenous nutrition may precipitate atrophy of intestinal mucosal villi, rendering children susceptible to cholestasis and related ailments. Our study reveals a markedly abbreviated duration of parenteral nutrition in the non-BPD group and a significantly prolonged period to reach total enteral feeding in the BPD group in comparison to the non-BPD group. This underscores the protective role of expeditious realization of total enteral feeding against BPD development. Hence, early implementation of enteral nutrition, contingent on conducive conditions, and expeditious removal of central venous catheters assumes paramount significance in BPD prevention and management.

It is important to note that there are complex interactions between nutrition and fluid management [36]. For example, a reduction in fluid supply may affect the availability of calories and other nutrients, while nutritional deficiencies may lead to difficulties in fluid management. Therefore, in the prevention and management of BPD, nutrition and fluid management cannot be considered in isolation, but need to be assessed in an integrated manner to develop an individualized management plan that balances nutritional supply with fluid load [37].

### Shortcomings and limitations of this meta-analysis

Compared to preceding meta-analyses [38], our study encompasses a more extensive array of pertinent research, integrating additional macronutrient impact indicators while imposing more stringent inclusion criteria and undertaking further subgroup analyses. Nevertheless, inherent limitations persist: (1) Discrepancies exist in prevailing clinical guidelines and consensuses on nutritional management of bronchopulmonary dysplasia in preterm infants both domestically and internationally. Divergent nutritional guidelines among the incorporated literature may yield clinical heterogeneity, potentially compromising systematic evaluation efficacy. (2) Adequacy of study effect indicators is not uniformly realized, necessitating statistical transformations for certain analyses.

## Conclusion

A higher intake of calories and macronutrients may be linked to a lower risk of bronchopulmonary dysplasia (BPD) in preterm infants. The timely initiation of enteral nutrition, particularly the early transition to total enteral feeding, could also help reduce BPD incidence. Early proactive enteral and parenteral nutrition is a key component in both prevention and management. A holistic approach to nutritional and fluid management, combined with optimized respiratory support strategies, may further contribute to lowering the risk of BPD development.

## Electronic supplementary material

Below is the link to the electronic supplementary material.

Supplementary Material 1

Supplementary Material 2

## Data Availability

The datasets generated during and analyzed during the current study are publicly available.

## Abbreviations

BPD: Bronchopulmonary dysplasia
CI: Confdence interval
