# Supplementary material for: Early nutritional management and risk of neonatal bronchopulmonary dysplasia: a systematic review and mata analysis
Source: Ital J Pediatr. 2025 Mar 24;51:94. doi: 10.1186/s13052-025-01929-5 (PMC11934726; doi:10.1186/s13052-025-01929-5)
Supplement: Supplementary file 1 — Supplementary Material 1 [file 13052_2025_1929_MOESM1_ESM.docx]

**search strategy**

| MeSH | Bronchopulmonary Dysplasia |
| --- | --- |
| Entry Terms: | pulmonary disease |
|  | Dysplasia, Bronchopulmonary |
|  | lung dysplasia |
|  | bronchial pulmonary dysplasia |
| MeSH | Infant, Premature |
| Entry Terms | Infants, Premature |
|  | Premature Infant |
|  | Premature Infants |
|  | Preterm Infants |
|  | Infant, Preterm |
|  | Infants, Preterm |
|  | Preterm Infant |
|  | Neonatal Prematurity |
|  | Prematurity, Neonatal |
| MeSH | Infant, Newborn |
| Entry Terms | Infants, Newborn |
|  | Newborn Infant |
|  | Newborn Infants |
|  | Neonate |
|  | Neonates |
|  | Newborns |
|  | Newborn |
| MeSH | Infant, Low Birth Weight |
| Entry Terms | Low-Birth-Weight Infant |
|  | Infant, Low-Birth-Weight |
|  | Infants, Low-Birth-Weight |
|  | Low Birth Weight Infant |
|  | Low-Birth-Weight Infants |
|  | Low Birth Weight |
|  | Low Birth Weights |
|  | Birth Weights, Low |
|  | Birth Weight, Low |
| MeSH | Parenteral Nutrition |
| Entry Terms | Nutrition, Parenteral |
|  | Intravenous Feeding |
|  | Feeding, Intravenous |
|  | Feedings, Intravenous |
|  | Intravenous Feedings |
|  | Parenteral Feedings |
|  | Feedings, Parenteral |
|  | Feeding, Parenteral |
|  | Parenteral Feeding |
| MeSH | Enteral Nutrition |
| Entry Terms | Nutrition, Enteral |
|  | Tube Feeding |
|  | Feeding, Tube |
|  | Enteral Feeding |
|  | Gastric Feeding Tubes |
|  | Feeding, Enteral |
|  | Force Feedings |
|  | Feedings, Force |
|  | Feeding, Force |
|  | Force Feeding |
|  | Tubes, Gastric Feeding |
|  | Tube, Gastric Feeding |
|  | Gastric Feeding Tube |
|  | Feeding Tubes, Gastric |
|  | Feeding Tube, Gastric |
| Entry Terms | nutrition |
|  | nutrient |
|  | nourishment |
| MeSH | Energy Intake |
| Entry Terms | Intake, Energy |
|  | Intake, Calorie |
|  | Calorie Intake |
|  | Caloric Intake |
| Entry Terms | liquid |
|  | fluid |
|  | fluid intake |
